# *Cblb* Gene Editing in T Cells Sustains Expansion and Immunogenic CAR T Tumor Killing Under Chronic Antigenic Stimulation

**DOI:** 10.3390/cells15151407

**Published:** 2026-08-03

**Authors:** Daniel Schreiber, Sebastian Peer, Christina Lutz-Nicoladoni, Jiří Koutník, Viktor Lang, Viana Wille, Isabel Hölzl, Dominik Humer, Nino Tokic, Dorothee Freimark, Mario Kuttke, Alexander Dohnal, Romana Gugenberger, Thomas Gruber, Nikolaus Thuille, Dominik Wolf, Victoria Klepsch, Kerstin Siegmund, Gottfried Baier

**Affiliations:** 1 Institute for Cell Genetics, Medical University of Innsbruck, 6020 Innsbruck, Austria; daniel.schreiber@i-med.ac.at (D.S.); sebastian.peer@i-med.ac.at (S.P.); viktor.lang@student.i-med.ac.at (V.L.); viana.wille@i-med.ac.at (V.W.); isabel.hoelzl@student.i-med.ac.at (I.H.); dominik.humer@dkfz-heidelberg.de (D.H.); nino.tokic@student.i-med.ac.at (N.T.); dorothee.freimark@i-med.ac.at (D.F.); thomas.gruber@i-med.ac.at (T.G.); nikolaus.thuille@i-med.ac.at (N.T.); victoria.klepsch@i-med.ac.at (V.K.); kerstin.siegmund@i-med.ac.at (K.S.); 2 Internal Medicine V, Hematology and Oncology, Comprehensive Cancer Center Innsbruck, Tyrolean Cancer Research Institute, Medical University of Innsbruck, 6020 Innsbruck, Austria; christina.lutz@i-med.ac.at (C.L.-N.); dominik.wolf@i-med.ac.at (D.W.); 3 Institute for Human Genetics, Medical University of Innsbruck, 6020 Innsbruck, Austria; 4 invIOs GmbH, Campus-Vienna-Biocenter, 1030 Vienna, Austria; mku@invios.com (M.K.); ado@invios.com (A.D.); rgu@invios.com (R.G.)

**Keywords:** CAR T cells, *Cblb* gene, chronic antigenic stimulation, immunogenic cell death, solid tumor microenvironment

## Abstract

**Highlights:**

**What are the main findings?**

- *Cblb* deficiency or transient *Cblb* silencing boosts endogenous and adoptive anti-tumor immunity in fully immunocompetent MC-38, MMTVneu and Panc02-EpCAM models.
- *Cblb*-deficient CAR T cells maintain expansion, effector function and tumor control under chronic antigen and TGF-β stress and induce increased pyroptotic tumor cell death.

**What are the implications of the main findings?**

- CBL-B is a cell-intrinsic checkpoint that limits T cell fitness, making it a target for more efficacious CAR T cell therapies in solid tumors.
- Targeting CBL-B can strengthen CAR T cell responses and promote more inflammatory, immunogenic cell death, potentially broadening anti-tumor immunity.

**Abstract:**

CBL-B is an intracellular E3 ubiquitin ligase that acts as a T cell checkpoint by raising activation thresholds and limiting effector function. Here, genetic targeting of CBL-B enhances the performance of adoptively transferred T cells and CAR T cells under tumor microenvironment-like stress. In fully immunocompetent mouse models, *Cblb* deficiency or transient *Cblb* silencing improves control of MC-38 colon carcinoma and autochthonous mammary tumors, demonstrating that CBL-B restrains anti-tumor immunity. *Cblb*-deficient T cells show enhanced expansion and effector/effector-memory differentiation during an in vivo mixed lymphocyte reaction, confirming a cell-intrinsic brake function of CBL-B during sustained antigenic challenge. In a syngeneic Panc02-EpCAM model, *Cblb*-deficient anti-EpCAM CAR T cells show superior tumor control, enhanced infiltration, prolonged survival, and preserved effector function despite chronic antigen exposure and TGF-β. Mechanistically, *Cblb* targeting maintains granzyme B and IFN-γ production and is associated in vitro with increased GSDME-linked pyroptotic tumor cell death, consistent with features of immunogenic cell death. These findings extend previous CBL-B CAR T work from lymphocyte-deficient to immunocompetent settings and support CBL-B inhibition as a strategy to engineer CAR T cells that resist suppressive tumor microenvironments while promoting a more inflammatory mode of tumor killing.

## 1. Introduction

Adoptive transfer of chimeric antigen receptor (CAR) T cells has transformed the treatment of selected hematologic malignancies, yet clinical efficacy in solid tumors remains limited. The discrepancy is largely attributable to the hostile tumor microenvironment (TME), which restricts T cell trafficking and imposes layered immunosuppression through inhibitory cytokines such as transforming growth factor-β (TGF-β), regulatory T cells (Tregs), myeloid-derived suppressor cells, metabolic stress, and chronic antigen exposure, which drive clonal T cell dysfunction. In parallel, antigen loss or downregulation under immunoediting pressure enables escape from monospecific CAR recognition and represents a major cause of relapse after adoptive cell therapy in both preclinical and clinical settings. Therefore, overcoming TME-driven dysfunction while simultaneously mitigating antigen escape remains a central challenge in extending the benefits of CAR T cells in the treatment of solid cancers [1,2,3].

The E3 ubiquitin ligase CBL-B has emerged as a critical intracellular immune checkpoint that raises T cell activation thresholds by ubiquitinating proximal TCR signaling intermediates, thereby constraining proliferation, cytokine production, and degranulation [4,5,6,7,8,9]. Genetic ablation of *Cblb* in mice enhances CD8^+^ T cell-mediated tumor control [10,11], confers relative resistance to Treg- and TGF-β-mediated suppression [12,13], and contributes to the suppressive program downstream of PD-1 engagement [14,15]. These properties have motivated *Cblb* targeting in engineered T cells: *Cblb*-deficient or *Cblb*-edited CAR T cells show reduced exhaustion, increased cytokine production, and superior tumor control in preclinical models [16,17], and small-molecule CBL-B inhibitors enhance T- and NK-cell function and CAR T manufacturing [17,18].

However, most prior work has been conducted in lymphocyte-deficient hosts, where enhanced anti-tumor activity primarily reflects direct CAR T cell cytotoxicity [16,17]. In such reductionist settings, the severely compromised adaptive immune compartment precludes assessments of whether *Cblb*-engineered CAR T cells can also recruit and amplify endogenous lymphocyte responses to improve tumor control. Moreover, it has remained unclear whether CBL-B inhibition can preserve CAR T effector function during prolonged antigen exposure and TGF-β-rich conditions that mimic solid tumor TMEs and whether *Cblb*-deficient CAR T cells influence the mode of tumor cell death, with potential consequences for immunogenicity and epitope spreading. These questions have not been directly addressed by previous *Cblb*-focused CAR T cell studies, which primarily examined short-term cytotoxicity and tumor control in lymphocyte-deficient hosts [16,17].

Immunogenic cell death (ICD) has been proposed as a route by which targeted therapies can prime de novo immune responses through the release of antigens and danger signals, particularly when cytotoxic lymphocytes trigger gasdermin-mediated pyroptosis in tumor cells [19,20,21,22]. In this context, we hypothesized that lowering the intracellular activation threshold via *Cblb* targeting could both render CAR T cells more refractory to TME-mediated suppression and preserve granzyme B-dependent mechanisms that convert apoptosis to GSDME-linked inflammatory pyroptosis, thereby promoting epitope spreading and mitigating antigen escape.

Here, using fully immunocompetent models, we show that *Cblb* targeting enhances endogenous and CAR-redirected anti-tumor immunity under TME-like stress, and it is associated with a qualitative shift toward GSDME-linked pyroptosis consistent with ICD. Thus, this study positions CBL-B not only as a regulator of CAR T cell magnitude but also as a candidate determinant of the inflammatory quality of tumor cell killing.

## 2. Materials and Methods

### 2.1. Mice

All mice, including C57BL/6NCrl (referred to hereinafter as WT, Charles River; RRID: IMSR_CRL:027), *Cblb*^−/−^ C57BL/6 (described previously in [9], kindly provided by J.M. Penninger (IMBA, Vienna, Austria)), FVB/N-Tg(MMTVneu)202Mul/J (referred to hereinafter as MMTVneuNtg, described previously in [23], Jackson Laboratory, RRID: IMSR_JAX:002376), B6.SJL-Ptprc^a^ Pepc^b^/BoyJ (referred to hereinafter as Ly5.1, Jackson Laboratory, RRID:IMSR_JAX:002014), and C57BL/6 × DBA/2 F1 hybrids (referred to hereinafter as B6D2F1, described previously in [24]) mice, were maintained under standard specific pathogen-free (SPF) conditions at the animal facility of the Medical University Innsbruck (approved as user by the Austrian Federal Ministry of Education, Science and Research, GZ: BMWF-66.011/0017-II/3b/2014). These conditions included an ambient temperature of 21–22 °C, a humidity level of 50%, a 12 h light/dark cycle, and ad libitum access to food and water. A total of 160 mice were used as recipients for in vivo experiments, while 54 mice were used as donors for CD3^+^ or CD8^+^ T cells. A maximum of five mice were housed per individually ventilated cage (IVC, Tecniplast, Murrhardt, Germany, Green line; 501cm^2^ floor area). Mice were monitored daily by the animal facility personnel and three times a week additionally during ongoing experiments for clinical signs of significant distress (using score sheets, e.g., taking the body condition score into account) by the scientists. Female mice were used for the tumor experiments. All studies and experimental protocols involving animals were reviewed and approved by the Animal Welfare Committee of the Medical University of Innsbruck and the Austrian Federal Ministry of Education, Science and Research (permit numbers: BMWF-66.011/0160-II/3b/2013, BMWF-66.011/0127-II/3b/2013, BM WF-66.011/0056-WF/V/3b/2017, 2020-0.345.526, 2023-0.203.973 & 2025-0.282.042). Experimental mice were selected at random from litters. At study endpoints or when reaching a defined humane endpoint (with death as an exception), mice were euthanized by gradual-fill carbon dioxide (CO_2_) inhalation in a euthanasia chamber (Vet-Tech Solutions, Congleton, UK, Double Cage Auto. CO_2_ Unit) using a displacement rate of 30% to 70% of the chamber volume per minute for five minutes; CO_2_ concentration was maintained for an additional five minutes and gradually exchanged with O_2_ for five minutes, a process automatically done by the device. This ensured sufficient CO_2_ levels after respiratory arrest. Death was confirmed by cervical dislocation. Tissues (tumor, spleen, lymph nodes, and blood) were collected immediately postmortem at defined time points. No anaesthesia was used for intravenous (i.v.), intraperitoneal (i.p.), or subcutaneous (s.c.) injections or blood sampling. The animals were restrained by hand for i.p. or s.c. injections, and animal restrainers were used (Sigma-Aldrich, St. Louis, MO, USA, Z756911) for i.v. injections. To reduce potential confounders, tumor injection, treatment, and tumor measurement were conducted in a randomized order. Allocation to experimental groups, tumor injection, and CAR T cell administration were performed by an investigator different from the blinded investigator who measured tumor size. Considerations of the 3Rs: The animals were handled by the same researcher whenever possible to minimize stress and help the mice become accustomed to that researcher. Furthermore, to achieve our goal of collecting as much relevant data as possible from a single animal and thereby minimizing the number of animals used, we worked closely together as a research group, particularly during the endpoint analyses.

### 2.2. Cell Lines and Cell Culture

Panc02 cells, a 3-methylcolanthrene-induced mouse pancreatic ductal adenocarcinoma (PDAC) cell line (described in [25,26], RRID: CVCL_D627) transduced to express murine EpCAM, were a kind gift from Professor Kobold (LMU, Munich, Germany). Retroviral transduction was employed with the pMP71-GFP construct (described in [27]) to generate Panc02-EpCAM cells stably expressing green fluorescent proteins (GFPs). Enrichment of GFP^+^ cells was achieved through flow cytometric sorting. Colon adenocarcinoma-derived MC-38 (RRID: CVCL_B288) cells were a kind gift from Professor Waldner (University of Erlangen, Erlangen, Germany). B16-F10-hEGFR cells were generated by lentiviral transduction of B16-F10 melanoma cells (ATCC, Manassas, VA, USA, CRL-6475, RRID:CVCL_0159) using the PWPXLD-EGFR-WT construct (previously described in [28], Addgene, Watertown, MA, USA, #133749, RRID:Addgene_133749), which was a kind gift from Chay Kuo. FACSort was used to select hEGFR-positive B16-F10 cells, which were then expanded and frozen in aliquots for future experiments. Murine tumor cells were cultivated in Dulbecco’s modified Eagle medium (DMEM, PAN-Biotech, Aidenbach, Germany, P04-01500) supplemented with 10% (*v*/*v*) fetal calf serum (FCS, Catus Biotech, Tutzing, Germany, BS-2020_500), 100 U/mL penicillin + 100 µg/mL streptomycin (PAN-Biotech, P06-7100), and 2 mM L-Glutamine (L-Glut, PAN-Biotech, P04-80050); in the following sections, it is referred to as DMEM+++. The packaging cell line PlatinumE (PlatE, Cell Biolabs Inc., San Diego, CA, USA, RV-101; RRID: CVCL_B488) was maintained in PlatE medium, which contains DMEM+++ supplemented with 10 µg/mL blasticidin (Sigma-Aldrich, 15205) and 1 µg/mL puromycin (Merck, Darmstadt, Germany, P8833) for selection of transgene-expressing cells. HEK293T cells (Abcam, Cambridge, UK, ab255449, RRID: CVCL_0063) were cultivated in DMEM+++. All cell lines were tested for the presence of mycoplasma (Invivogen, San Diego, CA, USA, rep-mys-20). For harvesting and passaging, adherent cells were washed once with 10 mL of warm 1× phosphate-buffered saline (PBS, Thermo Fisher Scientific, Waltham, MA, USA, J60236.K3). Then, 3 mL of TrypLE Express (Thermo Fisher Scientific, 12604021) was used to detach cells at 37 °C for two to five minutes. The enzymatic reaction was stopped using at least 6 mL of cell culture medium. The cell suspension was transferred into a 50 mL tube (Corning, Corning, NY, USA, BDL352070) and centrifuged for five minutes at 300× *g* and room temperature (RT). Subsequently, cells were used for experiments or resuspended in cell culture medium and split depending on the intended application. Typically, PlatE and HEK293T cells were split at a ratio of 1:5 while Panc02, B16-F10, and MC-38 cells were split at 1:10. All cell lines were cultivated in T-75 (Sarstedt, Nümbrecht, Germany, 83.3911.002) or T-175 flasks (Sarstedt, 83.3912.002) at 37 °C, 5% CO_2_, and a humidity level of 95%.

### 2.3. Isolation of Primary Mouse Splenocytes and T Cells

Subsequent to euthanasia, spleen and lymph nodes were extracted, and a single-cell suspension was prepared by mechanical disintegration using 100 µm strainers (Corning, 352360). Erythrocytes were removed by lysis for five minutes at RT using a red blood cell (RBC) lysis buffer (146 mM NH_4_Cl (Carl Roth, Karlsruhe, Germany, K298.1), 11.9 mM NaHCO_3_ (Sigma-Aldrich, S5761), and 161 µM ethylenediaminetetraacetic acid (EDTA, Carl Roth, X986.2) in ddH_2_O, pH 7.3) for all experiments except for the CD8^+^ T cells used in the breast cancer model. Following a wash and filter step using buffer C (1× PBS supplemented with 0.5% bovine serum albumin (BSA, Sigma-Aldrich, A7906) and 2 mM EDTA) and 40 µm cell strainers (Corning, 352340), viable cell counts were determined. Therefore, the cells were stained with acridine orange and propidium iodine (AO/PI) from the Cell Viability Kit (Biocat, Heidelberg, Germany, F2301-LG) and counted on a LUNA Automated Cell Counter (Logos Biosystems, Gunpo-si, Republic of Korea). CD3^+^ or CD8^+^ T cells were sorted by negative selection using MACS technology-based isolation kits: the pan-T cell isolation kit (Miltenyi Biotec, Bergisch Gladbach, Germany, 130-095-130), the CD8a^+^ T cell isolation kit (Miltenyi Biotec, 130-104-075), or the CD8a^+^ T cell isolation kit II mouse (Miltenyi Biotec, 130-095-236) used for preparation of donor cells in the breast cancer model. The cells were incubated with antibodies and magnetic beads according to the manufacturer’s protocol. CD3^+^ or CD8^+^ T cells were enriched by transferring the cell suspension through a 30 µm pre-separation filter (Miltenyi Biotec, 130-041-407) into LS columns (Miltenyi Biotec,130-042-401) that were placed in a QuadroMACS separator (Miltenyi Biotec, Germany). Columns were washed twice with buffer C, the flowthrough containing T cells was collected, and isolated T cells were counted, as described above. Sort purity was confirmed by flow cytometry.

### 2.4. Mixed Lymphocyte Reaction (MLR) Model: P -> F1

MLR experiments were conducted as established by Koutník et al. [24]. In summary, CD3^+^ T cells were isolated from C57BL/6NCrl, referred to as WT, or *Cblb*^−/−^ C57BL/6 mice (both CD45.2^+^, MHC haplotype b) as described above and labeled with cell proliferation dye eFluor670 (eBiosciences, San Diego, CA, USA, 65-0840). Therefore, T cells were washed twice with a Hanks buffered salt solution (HBSS) containing Mg^2+^ and Ca^2+^ (HBSS++, PAN-Biotech, P04-32505), adjusted to 1 × 10^7^ cells/mL in HBSS++, and an equal volume of a twofold labeling solution (10 µM proliferation dye in HBSS++) was added. Following five-minute incubation at 37 °C in a dark environment, the reaction was terminated with cold FCS and RPMI-1640 (PAN-Biontech., P04-17500). Thereafter, a wash step was performed with RPMI-1640 and, subsequently, with HBSS++. The cells were resuspended in HBSS++ (8 × 10^6^/100 µL), and 100 µL of the cell suspension was injected i.v. into the lateral tail vein (needle size 30 G) of B6D2F1 hybrid recipients (CD45.1^+^/2^+^, MHC haplotype b/d). Both male and female mice were used in the experiment, and the cells used in the adoptive cell transfer (ACT) were matched by sex. The maximum duration of the experiment was eight days to ensure that no clinical signs of graft-versus-host disease (GvHD) were experienced. Until the primary analyses were completed, the experimenters did not know which animals had received which genotype (blinded experimental approach).

Spleens from recipient mice were harvested and weighed on days 3 and 8 after ACT. For analyses of transferred T cells in the blood, mice were bled from the facial vein using a lancet into 1.5 mL microtubes (Sarstedt, 72.690.001) containing 3.5 µL of sodium-heparin (5000 IU/mL, Gilvasan Pharma, Vienna, Austria). A single-cell suspension of spleen tissue was obtained as described above. After lysis of erythrocytes in spleen cell suspensions and blood (as mentioned above), samples were analyzed by flow cytometry. Up to 2 × 10^6^ cells derived from spleens and 40 µL of blood samples were transferred into a 96-well round-bottom plate (Corning, 353077). The cells were washed with cold buffer C and incubated for five minutes with 3 µL of Fc-Block (1:10 in buffer C, clone: 2.4G2, BD Biosciences, San Jose, CA, USA, 553142, RRID: AB_394656) at 4 °C. Afterwards, samples were stained for 30 min at 4 °C in 40 µL of buffer C supplemented with antibodies for staining of surface antigens. These antibodies included the following: αCD4 V500 (1:100, clone: RM4.5, BD Biosciences, 560782, RRID: AB_1937327), αCD44 PerCp-Cy5.5 (1:100, clone: IM7, BioLegend, San Diego, CA, USA, 103032, RRID: AB_2076204), αCD45.1 Pacific Blue (1:100, clone: A20, BioLegend, 110722, RRID: AB_492866), αCD45.2 FITC (1:100, clone:104, BioLegend, 109806, RRID: AB_313443), and αCD62L APC-Cy7 (1:100, clone: MEL-14, BioLegend, 104428, RRID: AB_830799). Following an additional washing step with buffer C, expression of surface antigens was assessed on a flow cytometer. No data points obtained from mice were excluded in endpoint analyses.

### 2.5. siRNA Treatment of CD8^+^ T Cells

Subsequent to isolation, 1 × 10^7^ CD8^+^ T cells were subjected to electroporation using the Amaxa Nucleofector 2b device (Lonza, Basel, Switzerland) and the Mouse T Cell Nucleofector Kit (Lonza, VPA-1006) to ensure sufficient delivery of 1.5 µM of short interfering RNA (siRNA) targeting *Cblb* (ON-TARGETplus siRNA, Thermo Scientific Dharmacon, Lafayette, CO, USA, J-051830-05) or without a target (ON-TARGETplus Non-targeting Control siRNA, Thermo Scientific Dharmacon, D-001810-01). Transfection was conducted according to the manufacturer’s protocol employing the X-01 program designed for murine CD8^+^ T cells. Following transfection, cells were rested for a period of one hour (37 °C, 5% CO_2_) in RPMI-1640 supplemented with 5% (*v*/*v*) FCS and 2mM L-Glut. Thereafter, cells were used for ACT into MMTVneuNtg mice. To assess knockdown efficiency, CD8^+^ T cells nucleofected with *Cblb* siRNA or non-targeting control siRNA under conditions used for ACT were lysed 24 h after nucleofection and subjected to SDS–PAGE and immunoblotting as described below.

### 2.6. Breast Cancer Model

Four-month-old female MMTVneuNtg were palpated twice a week to detect spontaneous tumor development, typically occurring after four to twelve months. Three days after tumor detection, the animals were randomly allocated to groups and injected i.p. with 10 mg/kg Doxorubicin (Sandoz, Basel, Switzerland) followed by an ACT with nucleofected (siRNA silenced: non-targeting control or *Cblb* siRNA) and rested CD8^+^ T cells. Therefore, 5 × 10^6^ T cells (in 200 µL PBS) were injected i.v. into the lateral tail vein (30 G needle) at days 3, 10, and 17 after Doxorubicin injection. Assessment of tumor growth and weight was conducted two times a week using a caliper by scientists who were blinded to group allocation until completion of the primary analyses. Tumor size was estimated using the following equation, assuming a spheroidal shape: $V=\frac{1}{2}\times (length\times {width}^{2})$. Multiple tumors were summed up to one size. Mice were euthanized upon reaching predetermined humane endpoint criteria (based on an approved score sheet), including a maximum of 20% weight loss, a maximum tumor size of 1000 mm^3^, or decline in physical conditions. All data obtained from the mice were analyzed.

### 2.7. Constructs and Plasmids

For the production of the second-generation CAR targeting EpCAM (previously described in [28]), a pMP71 backbone containing the CAR construct was utilized (provided by C. Baum, Hannover, described in [25,26]). For the second CAR model, a CAR targeting human EGFR on a MSCV backbone was used (purchased from Vectorbuilder, Chicago, IL, USA). Both CARs consist of a single-chain variable fragment (scFv) (EGFR: Cetuximab, EpCAM: clone G8.8) fused to a CD8 hinge and CD28 transmembrane region. The intercellular domains consist of a CD28 co-stimulatory domain (amino acids 151-218, UniProt entry P31041) and a CD3zeta stimulatory domain (amino acids 52-164, UniProt entry P24161). Detection of the αEpCAM CAR was achieved through the usage of an myc tag positioned between the scFv and the hinge region or by expression levels of the fluorescent protein GFP, which was separated from the construct by a P2A self-cleavage sequence. The αhEGFR CAR was detected using biotinylated recombinant EGFR (rEGFR, Creative Biolabs, Shirley, NY, USA, CARD-LX025) and Streptavidin Bv421 (BioLegend, 405226). In this project, the pMP71-CAR plasmid was used for in vitro assays, while the pMP71-CAR-GFP construct was utilized in in vivo experiments. For transduction of Panc02 EpCAM cells with GFP, the pMP71-GFP plasmid (previously described in [27]) was used, while the PWPXLD-EGFR-WT plasmid was used to generate B16-F10-hEGFR (previously described in [28]).

### 2.8. Retrovirus Production

PlatE cells were split three times a week for a maximum period of 12 weeks after thawing, thereby establishing optimal conditions for viral particle production. Then, 8 × 10^5^ cells were seeded into each well of a tissue-culture-treated 6-well plate (Corning, 353046) in PlatE medium and cultured overnight. On the next day, upon reaching a cell confluency of 70–80%, the PlatE medium was replaced by DMEM supplemented with 2 mM L-Glut and 10% (*v*/*v*) FCS, without antibiotics. Introduction of plasmid DNA into cells was accomplished using the calcium phosphate precipitation method. For each well, 18 µg of transfer plasmid (pMP71-CAR-GFP or pMP71-CAR) and 15 µL of a calcium chloride solution (2.5 M CaCl_2_ (Sigma-Aldrich, C1016) in ddH_2_O) were mixed with ddH_2_O to a final volume of 150 µL. The plasmid solution was added, dropwise and under constant vortexing, to 150 µL of the transfection buffer (275 mM NaCl (Merck, 106404), 10 mM KCl (Merck, 104936), 42 mM 4-(2-Hydroxyethyl)-1-piperazineethanesulfonic acid (HEPES, Sigma-Aldrich, H3375), and 3.5 mM Na_2_HPO_4_ (Carl Roth, 4984.2) in ddH_2_O, pH 7.1). Subsequent to an incubation period of 30 min at RT, 300 µL of transfection mix was added to each well of PlatE cells. After 6 h of incubation at 37 °C and 5% CO_2_, the medium was replaced by DMEM+++, and cells were incubated for 42 h under the conditions mentioned above.

### 2.9. T Cell Activation, CAR Transduction, and Detection

Tissue culture-treated 12-well plates (Corning, 353225) were coated with 5 µg/mL of αCD3 antibody (clone: 145-2C11-CP082, BioXCell, New Haven, CT, USA, CP082) in 1× PBS overnight at 4 °C. On the following day, wells were washed twice with 1×PBS. Isolated CD8^+^ T cells were resuspended at a concentration of 3 × 10^6^/mL in CAR RPMI (RPMI-1640 supplemented with 10% (*v*/*v*) FCS, 100 U/mL penicillin, 100 µg/mL streptomycin, 2 mM L-Glut, 1 mM sodium pyruvate (Sigma-Aldrich, S8636), 1 mM HEPES (Sigma-Aldrich, H0887), and 50 µM 2-mercaptoethanol (Sigma-Aldrich, M3148)). The cell solution was supplemented with 10 ng/mL of human interleukin 2 (hIL-2, BioLegend, 589106) and 1 µg/mL of αCD28 antibody (clone: D665-CP042, BioXCell, CP042). Then, 3 × 10^6^ cells were seeded per well and incubated overnight at 37 °C, 5% CO_2_.

Non-tissue culture-treated 24-well plates (Corning, 351147) were incubated with 12.5 µg/mL Retronectin (Takara, Kusatsu, Japan, T100B) in 1× PBS overnight at 4 °C. The solution was removed the next day, and plate-bound Retronectin was blocked with 2% BSA fraction V (Carl Roth, T844.2) in ddH_2_O for 30 min at 37 °C. During the blocking period, the supernatant of transduced PlatE cells, containing viral CAR particles, was harvested and filtered through a 0.45 µm cellulose-acetate filter (Satorius AG, Göttingen, Germany, 17598-K). Fresh DMEM+++ was added to the cells for an additional harvesting step after 24 h. Following blocking, plates were washed with 1× PBS supplemented with 25 mM HEPES, and 2 mL of viral supernatant was spun on bound Retronectin at 3000× *g* for 2 h at 4 °C. During centrifugation, activated T cells were harvested, counted, and adjusted to 1 × 10^6^ cells/mL in CAR RPMI, which was supplemented with 10 ng/mL hIL-2 and αCD3/CD28 dynabeads (Thermo Fisher Scientific, 11453D) at a 1:1 bead to cell ratio (25 µL bead solution/mL). Subsequent to centrifugation, viral supernatant was replaced by 1 mL T cell solution, plates were centrifuged at 800× *g* for 30 min at 32 °C and incubated overnight at 37 °C, 5% CO_2_. On the next day, a second round of spinoculation was performed. Viral supernatants from PlatE cells were harvested and filtered as described above. Then, 500 µL of the virus-containing solution was mixed with an equivalent amount of CAR RPMI and added to the incubated T cells. Plates were centrifuged a second time at 800× *g* for 30 min at 32 °C and incubated. After 5 h, cells were harvested, activation beads were removed using a strong magnet, and T cells were counted. T cells were adjusted to 1.5 × 10^6^/mL in CAR RPMI containing 10 ng/mL interleukin 7 (IL-7, BioLegend, 577804) and 10 ng/mL interleukin 15 (IL-15, BioLegend, 566302). Cell concentrations were maintained at a constant level using CAR RPMI supplemented with 10 ng/mL IL-7/IL-15. Four days after the addition of viral supernatants, transduction efficiency of αEpCAM CAR was determined by flow cytometry by using expression of GFP or an αc-myc FITC antibody (1:400, clone: SH1-26E7.1.3, Miltenyi Biotec, 130-116-485, RRID: AB_2751321).

### 2.10. Lentiviral and Retroviral Transduction of Tumor Cells

Panc02-EpCAM-GFP cells were generated as previously described in [28]. In detail, PlatE cells were transfected with 18 µg of the pMP71-GFP plasmid, as mentioned above. One day before, 2 × 10^5^ Panc02-EpCAM cells were seeded into wells of a 6-well plate in DMEM+++ and incubated overnight. Shortly before transduction, the medium was replaced with DMEM+++ supplemented with 8 µg/mL polybrene (Sigma-Aldrich, TR-1003-G). Then, 2 mL of the filtered viral supernatant was added, and cells were spun at 800× *g* at 32 °C for 30 min. After centrifugation, cells were incubated overnight. On the next day, the cell medium was replaced with DMEM+++, and GFP expression of Panc02-EpCAM-GFP cells was determined using flow cytometry. In the following days, EpCAM-positive GFP-positive cells were enriched using flow sorting. Therefore, cells were harvested, and up to 1 × 10^7^ cells were stained in 100 µL of buffer C containing an αEpCAM APC antibody (1:400, clone: caa7-9G8, Miltenyi Biotec, 130-123-810, RRID: AB_2819524) for 20 min at 4 °C. Subsequently, cells were washed twice with cold buffer C and adjusted to 1 × 10^7^/mL in cold DMEM+++ supplemented with 2 mM EDTA. Directly before sorting, 1.1 µM of 4′,6-diamidino-2-phenylindole (DAPI, BioLegend, 4422801) was added for viability staining. Enrichment of viable GFP^+^ cells, with high EpCAM expression, was conducted at a BD FACS Aria I or a BD FACSDiscover^TM^ S8 (both BD Biosciences, USA) at the Cytometry Unit of the Medical University Innsbruck. The sorted cells were collected in DMEM+++ and immediately seeded into a T-175 flask.

B16-F10-hEGFR was generated using lentiviral supernatants containing the EGFR construct. Then, 2 × 10^6^ HEK293T cells were seeded in 10 cm dishes (Thermo Fisher Scientific, 10212951) and incubated overnight at 37 °C, 5% CO_2_. Before transfection, the medium was replaced by DMEM supplemented with 10% FCS and 2mM L-Glut, without antibiotics. Cells were transfected with 18 µg of PWPXLD-EGFR-WT (Addgene, #133749) and third-generation lentiviral packaging plasmids (5.75 µg pHELP1(PLP1), 5.75 µg pHELP2(PLP2), and 3.45 µg pHELP3 (VSVG), Creative Biolabs, CART-027CL) per dish with Lipofectamine 2000 (Thermo Fisher Scientific, 11668019), as described in the manufacturer’s protocol. The supernatant was harvested 48 and 72 h later, and the concentration of viral particles was increased using Lenti-X^TM^ Concentrator (Takara, 631232), following the manufacturer’s description. Then, 2 × 10^5^ B16-F10 cells per well in a 6-well plate were incubated overnight (37 °C, 5%CO_2_). On the next day, the medium was replaced with 2 mL of DMEM+++ supplemented with 8 µg/mL polybrene, and 20 µL of concentrated lentiviral particles was added. B16-F10-hEGFR cells were spun at 800× *g* at 32 °C for 2h. The following days, transduction efficiency was analyzed using the αEGFR-AF647 antibody (1:400, clone: AY13 BioLegend, 352917, RRID: AB_2650983). Enrichment of EGFR-positive cells was conducted as described above, using the αEGFR-AF647 antibody.

### 2.11. Tumor Injection and ACT with T Cells/CAR T Cells

MC-38, Panc02-EpCAM, and B16-F10-hEGFR cells were maintained as described above. At least one day prior to the s.c. tumor cell injection, the mice were shaved on the right flank. To achieve a confluency of 70-80% on the day of injection and to ensure optimal fitness, the tumor cells were split at a ratio of 1:2 one day before the injection. For MC-38 experiments, tumor cells were adjusted to 4 × 10^5^/100 µL (low dose) or 2 × 10^6^/100 µL (high dose) in HBSS++, and 100 µL was injected s.c. into the right flank of 8- to 16-week-old C57BL/6NCrl or *Cblb*^−/−^ C57 BL/6 mice (needle size: 27 G).

Prior to injection, EpCAM expression of Panc02-EpCAM cells was analyzed by flow cytometry using the αEpCAM APC antibody (1:400, clone: caa7-9G8, Miltenyi Biotec, 130-123-810, RRID: AB_2819524). Panc02-EpCAM cells were adjusted to 7.5 × 10^5^/100 µL, and 100 µL of the cell suspension was injected s.c. into the right flank of 8- to 12-week-old immunocompetent female C57BL/6N mice (needle size: 27 G).

hEGFR expression of B16-F10-hEGFR cells was analyzed via flow cytometry using the αEGFR-AF647 antibody (1:400, clone: AY13 Biolegend, 352917, RRID: AB_2650983). B16 cells were adjusted to 8 × 10^5^/100 µL, and 100 µL of the cell suspension was injected s.c. into the right flank of 8- to 12-week-old immunocompetent female Ly5.1 mice (needle size: 27 G). Tumor growth was assessed three times a week using a caliper by scientists who were blinded to group allocation until completion of the primary analyses. To minimize variability with regard to the 3Rs, the same person was always responsible for measuring the tumor. Tumor size was estimated as described above. Four days later, the tumor-bearing mice were randomly assigned to different treatment groups based on their tumor size. This ensured that all groups had the same average tumor size before CAR treatment. Then, 5 × 10^5^ (αEpCAM) or 1 × 10^6^ (αhEGFR) WT or *Cblb*^−/−^ CD8^+^ CAR T cells were injected i.v. into the lateral tail vein of each mouse (100 µL, 30 G needle). Mice were euthanized upon reaching predetermined humane endpoint criteria (max tumor size of 500 mm^3^, weight loss of 20%, decline in physical appearance).

### 2.12. Cell Preparation for Analysis of Tumor-Infiltrating Lymphocytes (TILs)

On day 25, after tumor injection with subsequent ACT of WT or *Cblb*^−/−^ CD8^+^ CAR T cells (as described above), mice were euthanized. Tumors were isolated from mice, cut into small pieces, and digested for 45 min at 37 °C in 1×PBS supplemented with 1 mg/mL DNAse I (Roche, Basel, Switzerland, 11284932201), 2.5 mg/mL Collagenase D (Roche, 11088858001), 2% (*v*/*v*) FCS, and 2.5 mM MgCl_2_ (Promega, Madison, WI, USA, A351H). Samples were vortexed frequently, and after incubation, 0.01 M ETDA was added for another five minutes at 37 °C, 5% CO_2_. Subsequently, tumors were meshed through a 100 µm and 40 µm cell strainer. Following centrifugation at 300× *g* for 7 min at RT, cells were resuspended in RPMI-1640 and used for flow cytometry analyses.

Up to 2 × 10^6^ cells of draining lymph nodes and tumors were transferred into a 96-well round-bottom plate. Staining of surface antigens was performed as described above with the following antibodies: αCD3 Pacific Blue (1:100, clone: 17A2, BioLegend, 100214, RRID: AB_493645), αCD4 V500 (1:100, clone: RM4.5, BD Biosciences, 560782, RRID: AB_1937327), and αCD8α PerCP-Cy5.5 (1:100, clone: 53-6.7, BioLegend, 100734, RRID: AB_2075238) or αCD8α APC (1:100, clone: 53-6.7, BD Biosciences, RRID: AB_398527). Mice reaching endpoint criteria before d25, and for which the draining lymph node could not be reliably identified, were excluded from analyses.

### 2.13. Serial Killing Assay

Sorted Panc02-EpCAM-GFP^+^ cells were harvested and checked for sufficient EpCAM and GFP expression using flow cytometry prior to the assay. If deemed necessary, additional enrichment of EpCAM-high and GFP-positive cells was conducted with flow sorting. Then, 6 × 10^4^ Panc02-EpCAM^hi^-GFP^+^ cells were seeded in CAR RPMI into a tissue culture-treated 96-well flat-bottom plate (Corning, 353072) and cultured (37 °C, 5% CO_2_) for 4 h to allow tumor cells to adhere. On day 5 after viral transduction and following assessment of transduction efficiency, CAR T cells were adjusted to 3 × 10^6^ /mL in CAR RPMI. Then, 3 × 10^5^ αEpCAM CAR T cells were added to tumor cells at an effector to target ratio (E:T) of 5:1 in CAR RPMI alone or supplemented with 1 ng/mL recombinant mouse TGF-β1 (eBioscience, 14-8342-80). Plates were directly placed into an Incucyte^®^ S3 Live-Cell Analysis System (Satorius AG, Germany). Live cell imaging was performed by taking brightfield and fluorescence pictures at two-hour intervals using the 10× objective of the device. Co-cultures of immune and tumor cells were restimulated every 48 h with fresh target cells. Therefore, fresh Panc02-EpCAM^hi^-GFP^+^ cells were harvested and adjusted to 6 × 10^5^/mL in CAR RPMI with or without 1 ng/mL TGF-β1. Then, 100 µL of the medium was removed from the co-culture and replaced by 100 µL of fresh tumor cells. Restimulation was conducted one hour before the next scan to ensure sufficient sedimentation of fresh target cells. The cytotoxicity of CAR T cells was assessed by alterations in the GFP fluorescence signal. Green integrated intensity per mm^2^ was plotted relatively to the first scan after each restimulation and analyzed using the built-in analysis software—Incucyte^®^ GUI Software version 2023A Rev2 (Satorius AG, Germany).

For flow cytometric analysis of surface antigens and cytokines, GolgiPlug (1:1000, BD Bioscience, 555029) and GolgiStop (1:1500, BD Bioscience, 554724) were added to co-culture wells one day after the start of cultivation. After five hours of incubation at 37 °C, 5% CO_2_, cells were transferred to a 96-well round-bottom plate for FACS staining. Cells were washed with cold buffer C, and surface antigens were stained (as described above) using the following antibodies: αc-myc FITC (1:400, clone: SH1-26E7.1.3, Miltenyi Biotec, 130-116-485, RRID: AB_2751321), αCD44 PE Cy7 (1:100, clone: IM7, BioLegend, 103030, RRID: AB_830787), and αCD62L APC (1:100, clone: REA828, Miltenyi Biotec, 130-112-837, RRID: AB_2658860). Samples intended for intracellular cytokine staining were transferred into a 96-well round-bottom plate. For assessment of viability, cells were washed twice with HBSS++ and stained in 100 µL of a viability dye solution (Fixable Viability Stain 780 (BD Biosciences, 565388, RRID: AB_2869673) 1:2000 in HBSS++) for 10 min in the dark at RT. Subsequently, cells were washed with cold buffer C, and surface antigens were stained (as stated above) with the following antibodies: αc-myc FITC (1:400, clone: SH1-26E7.1.3, Miltenyi Biotec, 130-116-485, RRID: AB_2751321), and αCD8α Brilliant Violet 510 (1:100, clone: 53-6.7, BioLegend, 100751, RRID: AB_2561389). After staining, cells were washed with buffer C and fixed in a Fixation Buffer (BioLegend, 420801) for 20 min at RT. Subsequently, cells were washed twice with 1× Intercellular Staining Permeabilization Wash Buffer (Perm/Wash Buffer, BioLegend, 421002) for permeabilization of cells. Staining of cytokines was performed for 30 min at 4 °C using a cytokine staining mix. For this, the following antibodies were diluted in 1× Perm/Wash Buffer: αGranzyme B PE (1:100, clone: NGZB, eBioscience, 12-8898-809 RRID: AB_10853811) and αInterferon γ PE-Cy7 (1:100, clone: XMG1.2, BioLegend, 505826, RRID: AB_2295770). After incubation, cells were washed with buffer C and subjected to FACS analysis.

### 2.14. Gasdermin E Cleavage Assay and Immunoblotting

Then, 3 × 10^5^ Panc02-EpCAM^hi^-GFP^+^ cells were co-cultured with 1.5 × 10^6^ CAR T cells in CAR RPMI (E:T ratio of 5:1) in a tissue culture-treated 24-well plate (Corning, 353047). CAR T cells were restimulated every 48 h with 3 × 10^5^ fresh tumor cells and subsequently harvested on days 0, 2, 4, and 8 after an incubation period of four hours. Therefore, supernatant was collected in a 15 mL tube (Corning, 352099), and wells were washed with 1× PBS. Then, 400 µL of TrypLE Express was added to each well for three to five minutes at 37 °C. Enzymatic reaction was stopped with 800 µL CAR RPMI, and the cell suspension was added into a 15 mL tube. Cells were centrifuged at 300× *g*, 4 °C for 7 min and washed with cold 1× PBS. Subsequently, cells were lysed in a 30 µL RIPA buffer (Sigma-Aldrich, R0278) supplemented with 1× Halt protease and phosphatase inhibitors and 5 mM EDTA (both Thermo Fisher Scientific, 78444) for 30 min on ice. Lysates were centrifuged at 15,000× *g* at 4 °C for 15 min, and the supernatant was transferred into a 1.5 mL tube containing a 7.5 µL Pierce™ Lane Marker Reducing Sample Buffer (Thermo Fisher Scientific, 39000). Proteins were heated to 95 °C for five minutes and separated via SDS-PAGE in a 4–12% polyacrylamide gel (Merck, MP41G10) for subsequent immunoblotting. After transfer of proteins (90 min, 1 mA/cm^2^) onto a polyvinylidene difluoride (PVDF, Merck, IPVH00010) membrane, the membrane was washed with 1× Tris-buffered saline and Tween (TBS-T, 10 mM Tris (Carl Roth, 4855.2), 150 mM NaCl and 0.05% (*v*/*v*) Tween (Sigma-Aldrich, P1379) in ddH_2_O, pH 8.0) and blocked with 5% (*w*/*v*) powdered milk (Carl Roth, T145.2)) in 1× TBS-T. Proteins were detected using the following primary antibodies diluted in 5% milk/TBS-T: αDNFA5/GSDME (1:1000, Abcam, clone: EPR19859, ab215191, RRID: AB_2737000) and αbeta-actin (1:1000, Santa Cruz Biotechnology, Dallas, TX, USA, clone: C4, sc-47778, RRID: AB_626632). After overnight incubation on a shaker (80 rpm) and 4 °C, secondary αmouse (1:5000, Thermo Fisher Scientific, 31430, RRID: AB_228307) or αrabbit (1:5000, Thermo Fisher Scientific, 31460, RRID: AB_228341) antibodies were added to the membrane for one hour at RT on a shaker (80 rpm). Subsequently, the membrane was washed with 1× TBS-T, and proteins were detected with Pierce^TM^ ECL Western Blotting Substrate (Thermo Fisher Scientific, 32106). Chemiluminescent signals were detected using a Fusion FX-7 (Vilber Lourmat, Collégien, France). Quantification of bands was done using ImageJ v1.51 (NIH, Bethesda, MD, USA).

Confirmation of siRNA-induced knockdown of CBL-B in CD8^+^ T cells or germline-based knockout in CAR T cells via immunoblot was conducted as described above, using the anti-CBLB antibody (1:1000, Abcam, clone: 246C5a, ab54362, RRID: AB_879652). For SDS PAGE and immunoblotting of siRNA-treated CD8^+^ T cells, the PageRuler^TM^ Prestained Protein Ladder (Thermo Fisher Scientific, 26616) was used as a marker.

### 2.15. Flow Cytometry

Flow cytometric analysis of stained samples, including samples from spleen, blood, draining lymph node, tumor, and in vitro cultures, was performed on a FACS Canto II (4-2-2 configuration, BD Biosciences, USA). Data was processed using the FlowJo software version 10.10.0 (FlowJo LLC, Ashland, OR, USA, RRID:SCR_008520).

### 2.16. Statistical Analysis

Data are represented as mean ± SEM. Statistical significance was calculated using repeated-measures two-way ANOVA (with Geisser–Greenhouse corrections applied to adjust for violations of sphericity assumption) for tumor growth curves, log-rank test for survival analyses, and multiple unpaired *t*-tests (with Holm–Šídák corrections for multiple testing and Welch corrections to account for unequal variances between groups) for pairwise comparisons. No further statistical assessment of normality was conducted. The experimental unit is the individual animal; for tumor size analyses, each tumor is considered an experimental unit. The exact number of experimental units per group is provided within each figure legend. Sample sizes were chosen to ensure robust data acquisition; however, no formal statistical method was used to predetermine sample size. Randomization was not based on a formal statistical method. Analyses were performed using GraphPad Prism v10.5.0 (GraphPad Software Inc, Boston, MA, USA). *p* values < 0.05 were considered statistically significant: * *p* ≤ 0.05, ** *p* ≤ 0.01, *** *p* ≤ 0.001, and **** *p* ≤ 0.0001.

## 3. Results

### 3.1. Cblb Deficiency Enhances Anti-Tumor Immunity in Preclinical Solid Tumor Models

**Figure 1:**
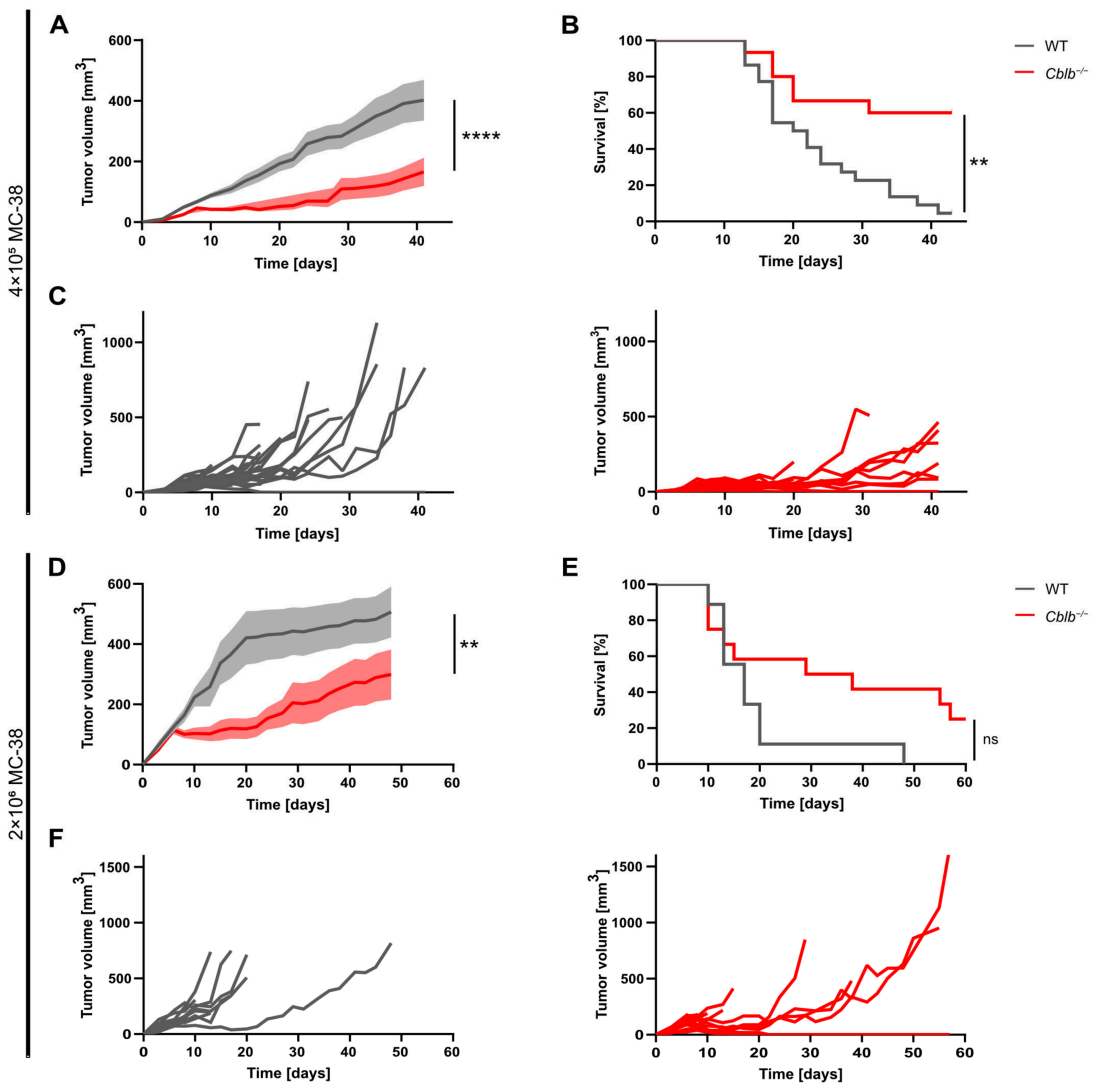
*Cblb* deficiency reduces tumor growth and prolongs survival in a subcutaneous MC-38 model. Wild-type (WT, in grey) and *Cblb*-deficient (*Cblb*^−/−^, in red) mice received 4 × 10^5^ (low dose, (**A**–**C**)) or 2 × 10^6^ (high dose, (**D**–**F**)) MC-38 cells via subcutaneous injection. (**A**) Average tumor growth curve of two independent experiments (*n* = 22 for WT and 15 *Cblb*^−/−^). (**B**) Survival curve of WT and *Cblb*^−/−^ mice. (**C**) Tumor growth of individual WT and *Cblb*^−/−^ mice in two independent experiments. (**D**) Average tumor growth curve of two independent experiments (*n* = 9 for WT and 12 for *Cblb*^−/−^). (**E**) Survival curve of WT and *Cblb*^−/−^ mice. (**F**) Tumor growth in single WT mice (grey) and *Cblb*^−/−^ mice (red) in two independent experiments. Statistical significance was determined using repeated measures two-way ANOVA with Geisser–Greenhouse correction (**A**,**D**) and log-rank test (**B**,**E**). Data are presented as mean ± SEM. ns = Not significant; ** *p* ≤ 0.01; **** *p* ≤ 0.0001.

To establish the therapeutic potential of *Cblb* targeting in solid tumors, we first evaluated germline *Cblb*^−/−^ mice in established syngeneic tumor models. Following subcutaneous injection of MC-38 colon carcinoma cells, *Cblb*^−/−^ mice exhibited significantly delayed tumor growth and prolonged survival compared to wild-type (WT) controls across both low-dose (4 × 10^5^ cells) and high-dose (2 × 10^6^ cells) MC-38 tumor challenge conditions (Figure 1A–F).

**Figure 2:**
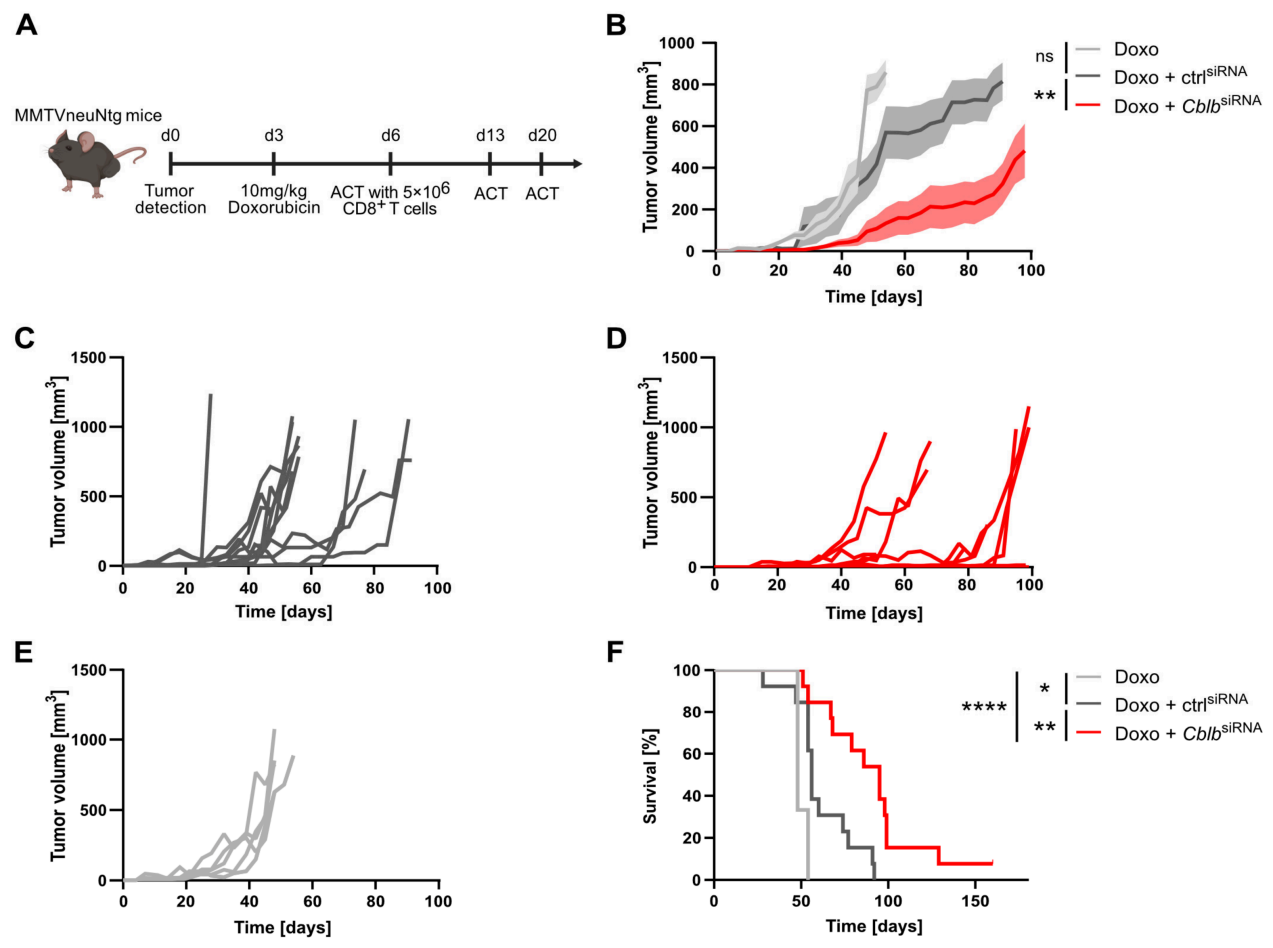
*Cblb*-silenced CD8^+^ T cells in combination with doxorubicin (Doxo) treatment show increased anti-tumor response and survival against MMTVneuNtg breast cancer. MMTVneuNtg mice were palpated twice weekly starting at the age of 4 months to detect early tumor development. Doxorubicin (10 mg/kg i.p.) was applied on d3 after tumor detection, followed by ACT of 5 × 10^6^ CD8^+^ T cells, control siRNA or *Cblb* siRNA nucleofected, respectively, on d3, d10, and d17 after Doxo injection. (**A**) Experimental setup and timeline. (**B**) Average tumor growth of mice treated with doxorubicin (light grey) or doxorubicin and control siRNA (dark grey) or *Cblb* siRNA (red) CD8^+^ T cells (*n*= 6 for Doxo only, 13 for both groups receiving an ACT). (**C**–**E**) Tumor growth of mice treated with doxorubicin only ((**C**), *n* = 6) and treated with doxorubicin and control siRNA CD8^+^ T cells. ((**D**), *n* = 13) Treated with doxorubicin and *Cblb* siRNA CD8^+^ T cells. ((**E**), *n* = 13) (**F**) Survival curve of mice in all three different treatment groups. Data represent four independent experiments (*n* = 6 for Doxo only, 13 for both groups receiving an ACT). Statistical significance was determined using repeated-measures two-way ANOVA with Geisser–Greenhouse correction (**B**) and log-rank test (**F**). Data are presented as mean ± SEM. ns= Not significant; * *p* ≤ 0.05; ** *p* ≤ 0.01; **** *p* ≤ 0.0001.

Building on these findings, which firmly validated CBL-B as a negative regulator of endogenous anti-tumor immunity, we next combined T cell-intrinsic *Cblb* targeting with a chemotherapy that increases tumor immunogenicity and evaluated its efficacy in an autochthonous breast cancer model. To this end, we switched from a germline knockout to a transient siRNA-mediated knockdown of *Cblb* in adoptively transferred CD8^+^ T cells and tested these cells in the MMTVneuNtg breast cancer model, which provides an autochthonous, HER2/neu-driven tumor with a suppressive, fibrotic niche (Figure 2A). Efficient CBL-B depletion in nucleofected CD8^+^ T cells under these conditions was confirmed by immunoblot analyses, showing reduced CBL-B protein levels in *Cblb* siRNA-treated cells compared to non-targeting controls (Supplementary Figure S1A).

*Cblb* knockdown in adoptively transferred CD8^+^ T cells, combined with doxorubicin treatment, resulted in enhanced tumor regression and improved survival in the autochthonous MMTVneuNtg breast cancer model compared to control siRNA-treated cells or doxorubicin alone (Figure 2B–F).

Together, these results confirm that CBL-B acts as a negative regulator of anti-tumor immunity in solid tumors and further demonstrate the feasibility of a therapeutic strategy in which *Cblb* is transiently targeted in CD8^+^ T cells prior to adoptive transfer. Notably, the use of an autochthonous HER2/neu-driven tumor model extends previous *Cblb* studies into a clinically more relevant immunocompetent setting and suggests that transient CBL-B suppression can potentiate therapy even within an established suppressive tumor niche.

### 3.2. Cblb-Deficient T Cells Exhibit Enhanced Allogeneic Activation In Vivo

**Figure 3:**
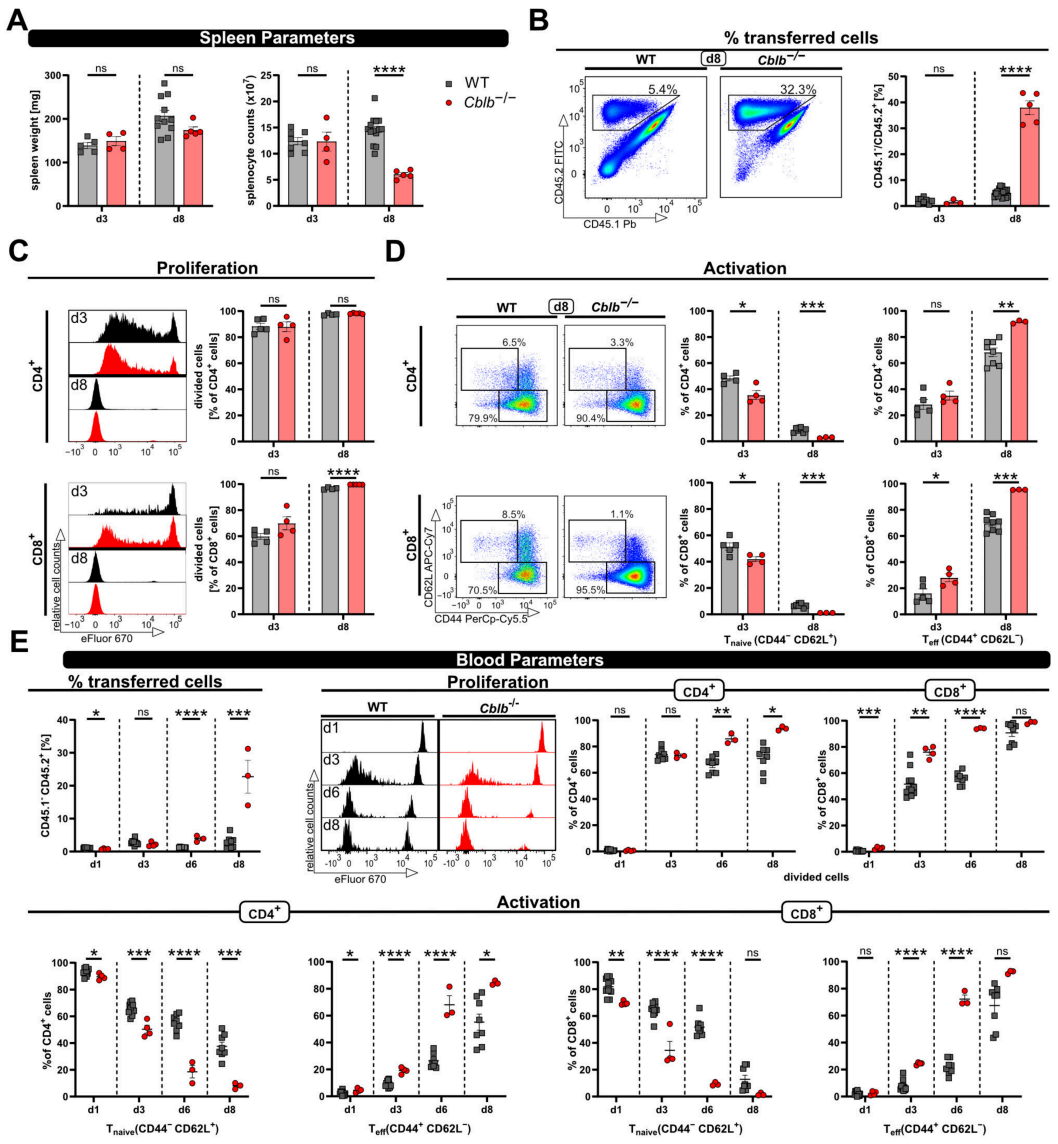
*Cblb*-deficient T cells show increased allogeneic activation and proliferation in a mixed lymphocyte reaction (MLR) model in vivo. In total, 8 × 10^6^ CD3^+^ T cells from WT or *Cblb*^−/−^ mice (both CD45.2^+^, MHC haplotype b) were transferred to the B6D2F1 hybrid (CD45.1^+^/2^+^, MHC haplotype b/d) recipients via i.v. injection. Transferred T cells from spleen and blood were analyzed with flow cytometry. (**A**) Spleen weight and splenocyte count at day 3 and 8 after ACT (*n* = 4–17, each dot represents data from one mouse). (**B**) Frequency of transferred T cells (CD45.2^+^) in the spleens of recipients (CD45.1^+^/2^+^) was determined on days 3 and 8 (*n* = 3–17, each dot represents data from one mouse). (**C**) Dilution of proliferation dye eFluor670 with each cell division was used to analyze proliferation of pre-stained, transferred T cells (WT in black/grey, *Cblb*^−/−^ in red; *n* = 4–5, each dot represents data from one mouse) (**D**) CD44 and CD62L were used to assess frequency of naive and effector/memory subsets among transferred CD4^+^ and CD8^+^ T cells by flow cytometry (*n* = 3–8, each dot represents data from one mouse). (**E**) Frequency, proliferation, and naive and effector/memory subsets of transferred T cells in the blood of recipient mice were analyzed as described above (**A**–**D**) (*n* = 3–11, each dot represents data from one mouse). Statistical significance was determined using multiple unpaired *t*-tests with Holm–Šidák and Welch corrections (**A**–**E**). Data are presented as mean ± SEM. Representative histograms or FACS dot plots of individual mice are shown. ns = Not significant; * *p* ≤ 0.05; ** *p* ≤ 0.01; *** *p* ≤ 0.001; **** *p* ≤ 0.0001.

To define the T cell-intrinsic mechanisms underlying the enhanced anti-tumor activity, particularly in an adoptive cell transfer (ACT) setting, we next examined the behavior of *Cblb*-deficient T cells in an in vivo mixed lymphocyte reaction (MLR) model, which allows analysis of T cell activation and differentiation in a physiological environment. On days 3 and 8 after ACT of CD3^+^ T cells (CD45.2^+^, C57BL/6 background) into semi-allogeneic B6D2F1 (CD45.1/2) recipients, spleen weight and cellularity were assessed, and the fate of transferred T cells was tracked by flow cytometry using CD45.1/2 congenic markers. Spleen weights were comparable between recipients of WT and *Cblb*^−/−^ T cells, but total splenic cell counts at day 8 were significantly lower in mice receiving *Cblb*-deficient T cells (Figure 3A). This occurred despite a markedly increased frequency of donor-derived *Cblb*-deficient T cells, indicating preferential expansion of the transferred population concomitant with loss of host lymphocytes (Figure 3B). This seemingly paradoxical pattern is consistent with the kinetics of this MLR model, in which early activation and proliferation of donor CD4^+^ T cells drive host B cell expansion, followed by the emergence of donor CD8^+^ T cells that progressively eliminate host lymphocytes. Our data suggest that this cytotoxic phase is accelerated when *Cblb*-deficient T cells are transferred.

In line with this, proliferation tracking by eFluor670 dilution revealed accelerated cell division of *Cblb*-deficient T cells (Figure 3C), and phenotypic analyses showed a pronounced skewing toward CD44^high^ effector and effector-memory subsets (Figure 3D). To assess activation kinetics in more detail, we analyzed peripheral blood at multiple time points after ACT. Differences in activation and differentiation between WT and *Cblb*-deficient T cells were already evident at day 3 and further amplified by day 6 post-ACT, pointing to accelerated activation and effector differentiation in the absence of CBL-B (Figure 3E).

Together, these findings indicate that CBL-B imposes a cell-intrinsic brake on T cell activation, expansion, and effector differentiation under conditions of sustained antigenic stimulation, helping to explain the superior effector responses and enhanced tumor control observed in the solid tumor models. More broadly, this in vivo MLR system provides physiologic evidence that *Cblb* deletion accelerates T cell outgrowth and effector/memory skewing during persistent challenge, thereby linking CBL-B directly to impaired T cell fitness in settings relevant to adoptive immunotherapy.

### 3.3. Genetic Cblb Deletion Enhances Anti-EpCAM CAR T Cell Efficacy in a Solid Tumor Model

**Figure 4:**
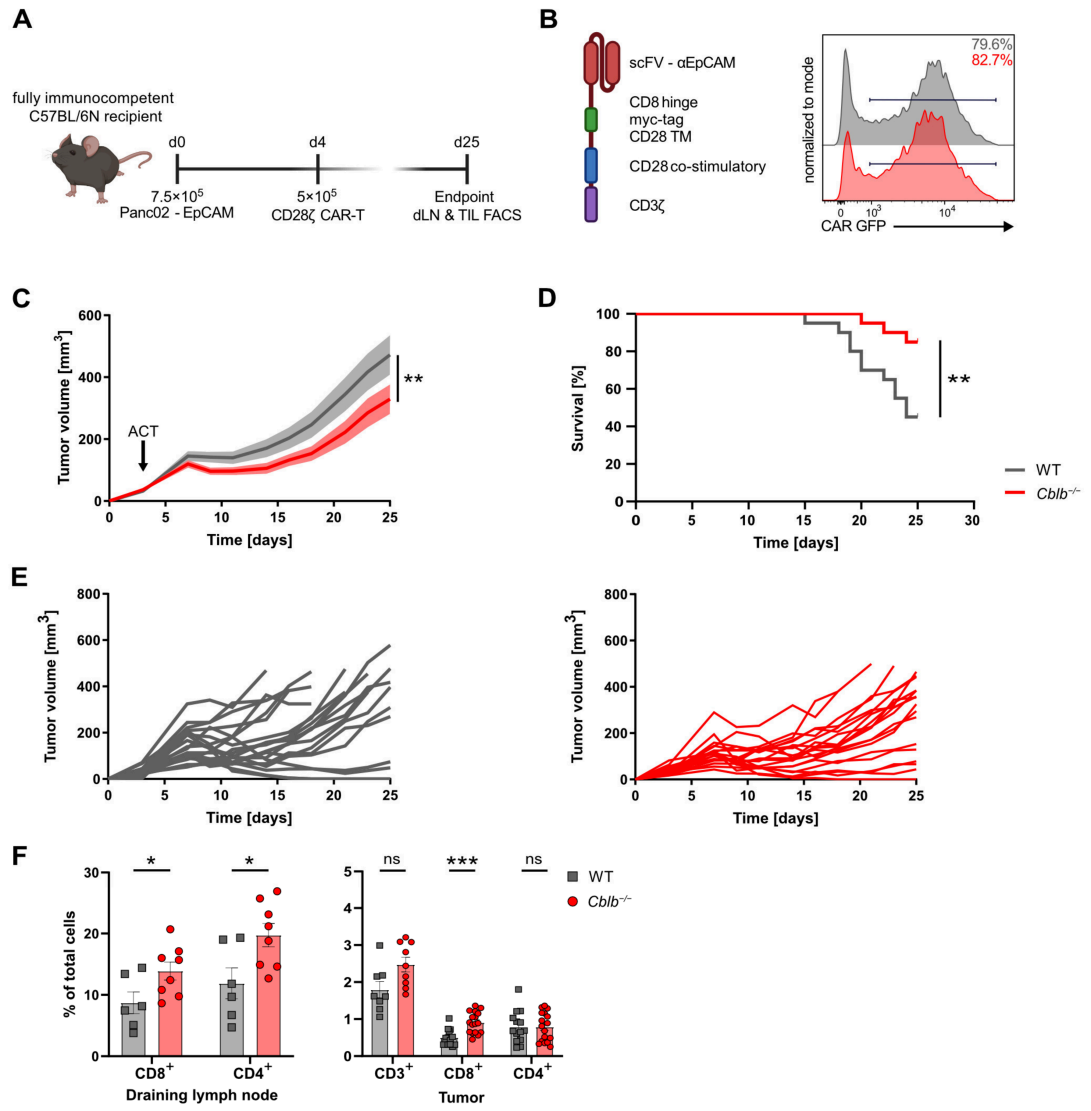
Therapeutic benefit of anti-EpCAM CAR T cells in the murine Panc02 tumor model by genetic *Cblb* inhibition. C57BL/6NCrl mice received 7.5 × 10^5^ EpCAM-expressing Panc02 (Panc02-EpCAM) tumor cells subcutaneously, and a therapeutic ACT of 5 × 10^5^ CD8^+^ CAR T cells was performed on d4 after tumor injection. Surviving mice were sacrificed on d25, and tumor-infiltrating lymphocytes (TILs) and draining lymph nodes (dLNs) were analyzed by flow cytometry. (**A**) Schematic timeline of experiment. (**B**) Second-generation CAR construct used for retroviral transduction and exemplary flow cytometric analysis of transduction efficiency in WT (grey) and *Cblb*^−/−^ (red) CD8^+^ T cells. Positive % CAR indicated in numbers. (**C**) Average tumor growth curve of two independent experiments (*n* = 20 for WT and 20 for *Cblb*^−/−^). (**D**) Survival curve of mice that received WT or *Cblb*^−/−^ CD8^+^ CAR T cells. Mice harboring tumors with a size > 500mm^3^ were sacrificed. (**E**) Tumor growth in mice that received WT (left) and *Cblb*^−/−^ (right) CD8^+^ CAR T cells in two independent experiments. (**F**) Frequency of CD3^+^, CD4^+^, and CD8^+^ T cells within the draining lymph node (left) and tumor (right). Data represent one (dLN, *n* = 6–8, each dot represents data from one mouse) and two independent experiments (TILs, *n* = 8–17, each dot represents data from one mouse). Statistical significance was determined using repeated-measures two-way ANOVA with Geisser–Greenhouse correction (**D**), log-rank test (**E**), and multiple unpaired *t*-tests with Holm–Šidák and Welch corrections (**F**). Data are presented as mean ± SEM. ns = Not significant; * *p* ≤ 0.05; ** *p* ≤ 0.01; *** *p* ≤ 0.001.

Building on these observations, we next evaluated *Cblb* targeting in the context of engineered CAR T cells in a syngeneic Panc02-EpCAM model in fully immunocompetent recipient mice (Figure 4A).

WT and *Cblb*^−/−^ CD8^+^ T cells were retrovirally transduced with a second-generation anti-EpCAM CAR construct containing CD28 and CD3ζ signaling domains (Figure 4B). Therapeutic transfer of *Cblb*-deficient CAR T cells delayed tumor growth and significantly improved survival compared with WT CAR T cells (Figure 4C–E). Flow cytometric analysis of tumors and draining lymph nodes (dLN) revealed higher frequencies of endogenous CD8^+^ and CD4^+^ T cells in the dLN and an increased CD8^+^ T cell fraction in the tumor tissue of the *Cblb*-deficient group (Figure 4F), indicating improved trafficking and/or local persistence of host T cells. Notably, CD8^+^ cells were selectively enriched, consistent with a CD4/CD8 shift within a stable CD3^+^ compartment that favors endogenous CD8^+^ effector cells in *Cblb*-deficient CAR T-treated tumors.

Additionally, we employed a second, independent murine CAR T cell model using a second-generation CAR construct (CD28 and CD3ζ signaling domains) targeting human EGFR (hEGFR) ectopically expressed on B16-F10 melanoma cells. In this additional model, mice treated with *Cblb*-deficient anti-hEGFR CAR T cells showed improved tumor control and survival compared to WT CAR T cell therapy (Supplementary Figure S2A–D).

Together with the MLR data, these results support the view that CBL-B imposes a cell-intrinsic limit on T cell activation, expansion, and effector function under conditions of strong and/or chronic antigen exposure and that its removal enhances CAR-directed T cell responses in solid tumors. Importantly, in contrast to earlier *Cblb*-engineered CAR T cell studies that were largely confined to lymphocyte-deficient hosts, these data demonstrate therapeutic benefit in a fully immunocompetent solid tumor model.

### 3.4. Cblb Deletion Sustains CAR T Cytotoxicity During Chronic Stimulation and TGF-β Exposure

**Figure 5:**
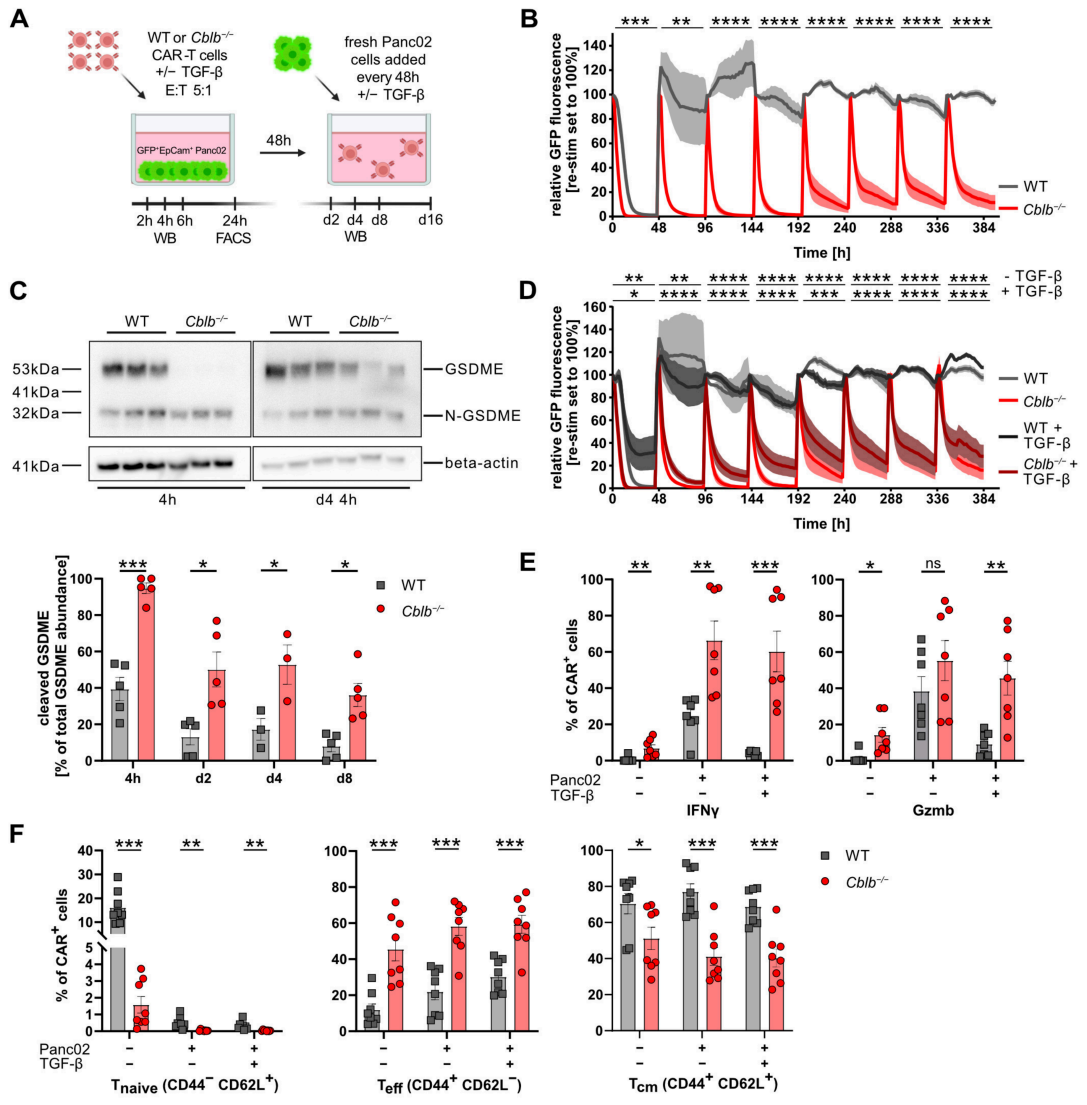
*Cblb* depletion in CAR T cells improves cytotoxicity and pyroptotic cell death in Panc02-EpCAM cells during chronic stimulation. Sorted GFP^+^ Panc02-EpCAM tumor cells were co-cultured with WT or *Cblb*^−/−^ CAR T at an effector to target (E:T) ratio of 5:1 to assess cytotoxicity during chronic stimulation in medium with or without TGF-β. Fresh tumor cells were added every 48 h at an E:T ratio of 5:1. Cytotoxicity was monitored by measuring the GFP signal of tumor cells over a period of 16 days. Gasdermin E cleavage was assessed after 4h and 4h after re-stimulation on days 2, 4, and 8 by immunoblotting. Flow cytometric analysis of CAR T cells was performed after 24h of co-culture. (**A**) Experimental setup of the serial killing assay. (**B**) Cytotoxicity of WT and *Cblb*^−/−^ CAR T cells. Data represent three independent experiments (*n* = 7 for WT and 7 for *Cblb*^−/−^). (**C**) Gasdermin E cleavage assay after each round of serial killing. Four hours after re-stimulation of WT or *Cblb*^−/−^ CAR T cells with Panc02-EpCAM tumor cells, GSDME cleavage in tumor cells was analyzed by immunoblotting. Quantification was performed using ImageJ. Data represent two independent experiments (*n* = 5 for WT and 5 for *Cblb*^−/−^, each dot represents data from one biological replicate), except for d4 (one experiment, *n* = 3 for WT and 3 *Cblb*^−/−^, each dot represents data from one biological replicate). (**D**) Cytotoxicity of WT and *Cblb*^−/−^ CAR T cells in medium with or without 1ng/mL TGF-β. Data represent two independent experiments (*n* = 5 for WT and 5 for *Cblb*^−/−^). (**E**) IFN-γ (left) and Granzyme B (right) production by CAR T cells after 24h of co-culture with tumor cells, analyzed by flow cytometry. Data represent three independent experiments (*n* = 7 for WT and 7 for *Cblb*^−/−^, each dot represents data from one biological replicate). (**F**) Differentiation status of CAR T cells after 24h of co-culture with tumor cells, analyzed by flow cytometry. Data represent four independent experiments (*n* = 8 for WT and 8 for *Cblb*^−/−^). Statistical significance was determined using repeated-measures two-way ANOVA with Geisser–Greenhouse correction (**B**,**D**) or multiple unpaired *t*-tests with Holm–Šidák and Welch corrections (**C**,**E**,**F**). Data are presented as mean ± SEM. ns = Not significant; * *p* ≤ 0.05; ** *p* ≤ 0.01; *** *p* ≤ 0.001; **** *p* ≤ 0.0001.

To model the chronic antigen exposure and immunosuppressive conditions characteristic of solid tumor TMEs, we developed a serial killing assay using live-cell imaging. GFP^+^ Panc02-EpCAM target cells were co-cultured with WT or *Cblb*^−/−^ CAR T cells at a 5:1 effector-to-target ratio, with fresh tumor cells added every 48 h over 16 days (Figure 5A). *Cblb*^−/−^ CAR T cells maintained significantly higher cytotoxicity across multiple restimulation cycles compared to WT cells (Figure 5B). When TGF-β was added to mimic TME immunosuppression, WT CAR T cytotoxicity was profoundly impaired, whereas *Cblb*^−/−^ CAR T cells remained highly effective, demonstrating near-complete resistance to TGF-β-mediated suppression (Figure 5D). Flow cytometric analysis confirmed that *Cblb*^−/−^ CAR T cells maintained elevated production of interferon-γ and granzyme B even in the presence of TGF-β, levels that were substantially reduced in WT cells under the same conditions (Figure 5E). Phenotypic analysis indicated retention of a more favorable differentiation profile in *Cblb*^−/−^ cells, with enrichment of effector and effector-memory subsets (Figure 5F).

Thus, *Cblb*-deficient CAR T cells combine durable cytotoxicity with preserved functional fitness during prolonged restimulation and suppressive cytokine exposure, highlighting a key advantage of CBL-B checkpoint targeting under chronic TME-like stress.

### 3.5. Cblb^−/−^ CAR T Cells Induce Pyroptotic Tumor Cell Death via GSDME Cleavage

The elevated granzyme B production by *Cblb*^−/−^ CAR T cells prompted investigation of the mode of tumor cell death. Immunoblot analysis of co-cultured tumor cells revealed robust cleavage of GSDME in target cells killed by *Cblb*^−/−^ CAR T cells but minimal cleavage by WT CAR T cells (Figure 5C). GSDME clipping was detectable as early as 4 h post-stimulation and persisted across serial restimulation cycles on days 2, 4, and 8, with significantly higher levels induced by *Cblb*^−/−^ CAR T cells at each time point. The cleaved N-terminal GSDME fragment (p30) is the executor of pyroptosis, forming membrane pores that cause inflammatory lytic cell death [22,29]. This shift toward more frequent pyroptotic tumor cell death suggests a qualitative change in the mode of killing, which may have important immunological consequences.

In principle, pyroptosis-driven lysis can release intracellular damage-associated molecular patterns (DAMPs) and tumor antigens into the extracellular space, thereby creating a pro-inflammatory milieu compatible with features of ICD. Our data therefore indicate that *Cblb* deletion not only enhances the magnitude of CAR T cell-mediated killing but is also associated with a change in its inflammatory character. While previous work has shown that CAR T cells can induce GSDME-dependent pyroptosis, these findings suggest that *Cblb* targeting may represent an intervention that biases killing toward a GSDME-linked pyroptotic phenotype across repeated rounds of stimulation. These observations are based on in vitro GSDME cleavage in co-cultured tumor cells, and the resulting implications for in vivo DAMP release, cross-presentation, and epitope spreading remain hypothetical and will require dedicated mechanistic studies. Nevertheless, this qualitative shift raises the possibility of epitope spreading and broader endogenous immune recruitment, which will require direct validation in future in vivo studies.

## 4. Discussion

Consistent with previous work, our data support CBL-B as a pivotal intracellular checkpoint that constrains CAR T cell activity in solid tumors by coupling T cell activation thresholds to dominant suppressive cues in the TME [12,13,16,17]. *Cblb* deletion enhances CAR T infiltration, persistence, and cytotoxic function, and it allows sustained interferon-γ and granzyme B production under chronic antigen exposure and TGF-β-rich conditions, which are hallmark features of solid tumor immune evasion that conventional CAR T cells rarely overcome [30,31,32]. In an in vivo mixed lymphocyte reaction, *Cblb*-deficient T cells exhibited greater expansion and effector/effector-memory differentiation under strong stimulation, underscoring a cell-intrinsic brake function that likely contributes to superior tumor control. In this way, our findings extend prior work on CBL-B by showing that lowering the activation threshold through *Cblb* targeting can help maintain effector programming and function in a suppressive microenvironment [10,33].

Beyond earlier studies that primarily assessed short-term CAR T activity in lymphocyte-deficient hosts, the present work demonstrates that *Cblb* targeting supports sustained T cell expansion and function in fully immunocompetent solid tumor models. A further mechanistic advance is the observation that *Cblb* targeting not only increases the magnitude of tumor killing but also appears to influence its quality. *Cblb*-deficient CAR T cells maintained high granzyme B levels and induced robust GSDME cleavage in target cells across serial restimulation cycles, consistent with a shift toward more frequent pyroptotic, inflammatory death. This shift in tumor cell death may have important immunological consequences. In principle, GSDME-linked pyroptosis can release DAMPs and tumor antigens into a pro-inflammatory milieu, a context known to favor, at least conceptually, cross-priming and epitope spreading [19,20,21,29,34]. Our observations raise the possibility that CBL-B controls not only whether CAR T cells kill efficiently but also how they kill, thereby shaping the immunogenic imprint left on the tumor bed.

These findings complement existing strategies to improve adoptive cell therapy in solid tumors. “Armored” CAR T designs that directly inhibit TGF-β pathways primarily aim to maintain effector function, whereas ICD-inducing chemo-, radio-, and virotherapies promote epitope spreading but lack antigen precision [21,34,35,36]. By contrast, *Cblb* targeting connects an intracellular checkpoint to both outcomes, namely TME resistance and more immunogenic killing, and therefore, it offers a unified approach to two central barriers in solid tumor immunotherapy. To our knowledge, this is the first study to connect intracellular checkpoint targeting with sustained GSDME-linked killing by CAR T cells under chronic restimulation, positioning CBL-B as a nodal regulator of both functional persistence and the inflammatory quality of tumor cell death.

At the same time, several limitations should be acknowledged. Our conclusions regarding ICD and epitope spreading are based on in vitro GSDME cleavage and indirect in vivo readouts and thus remain inferential rather than definitive. Further work will be required to establish mechanistic causality and to evaluate safety, including the risk that more inflammatory killing could exacerbate cytokine release or tissue damage. In addition, interspecies differences in gasdermin family usage highlight the need to confirm the relevance of this pathway in human tumor models.

In summary, our work supports a model in which *Cblb*-targeted adoptive cell therapy does not merely amplify cytotoxicity but retunes effector–TME interactions while shifting tumor cell death toward a more inflammatory mode. This combination of TME-resistant programming and ICD-compatible killing provides a preclinical rationale for translating *Cblb*-engineered CAR T products and, ultimately, for integrating them with microenvironment remodeling to convert otherwise “cold” solid tumors into sites capable of sustaining broader, anti-tumor immunity.

## Data Availability

The original contributions presented in this study are included in the article. Further inquiries can be directed to the corresponding author. The raw data supporting the conclusions of this article will be made available by the authors upon request.

## Supplementary Materials

The following supporting information can be downloaded at https://www.mdpi.com/article/10.3390/cells15151407/s1. Figure S1: Confirmation of Cblb knockdown or knockout in siRNA treated CD8^+^ T cells and germline CAR T cells; Figure S2: Therapeutic benefit of anti-hEGFR CAR T cells in murine B16-F10 tumor model by genetic *Cblb* inhibition.

## Abbreviations

The following abbreviations are used in this manuscript:

ACT	Adoptive cell transfer;
AO/PI	Acridine orange/propidium iodine;
BSA	Bovine serum albumin;
Ca^2+^	Calcium ion;
CaCl_2_	Calcium chloride;
CAR	Chimeric antigen receptor;
CBL-B	Casitas B-lineage lymphoma proto-oncogene B;
CO_2_	Carbon dioxide;
DAMP	Damage-associated molecular pattern;
DAPI	4′,6-diamidino-2-phenylindole;
dLN	Draining lymph node;
DMEM	Dulbecco’s modified Eagle medium;
Doxo	Doxorubicin;
EDTA	Ethylenediaminetetraacetic acid;
EGFR	Epidermal growth factor receptor;
EpCAM	Epithelial cell adhesion molecule;
E:T	Effector-to-target ratio;
FACS	Fluorescence-activated cell sorting;
FCS	Fetal calf serum;
GFP	Green fluorescent protein;
GSDME	Gasdermin E;
GvHD	Graft-versus-host disease;
GzmB	Granzyme B;
hEGFR	Human epidermal growth factor receptor;
HEPES	4-(2-Hydroxyethyl)-1-piperazineethanesulfonic acid;
HBSS	Hanks buffered salt solution;
hIL-2	Human interleukin-2;
ICD	Immunogenic cell death;
IFN-γ	Interferon γ;
IL-7/IL-15	Interleukin 7 or 15;
i.p.	Intraperitoneal;
IVC	Individually ventilated cage;
i.v.	Intravenous;
KCl	Potassium chloride;
L-Glut	L-Glutamine;
Mg^2+^	Magnesium ion;
MgCl_2_	Magnesium chloride;
MHC	Major histocompatibility complex;
MLR	Mixed lymphocyte reaction;
NaHCO_3_	Sodium bicarbonate;
Na_2_HPO_4_	Disodium hydrogen phosphate;
NH_4_Cl	Ammonium chloride;
NIH	National Institutes of Health;
NK cell	Natural killer cell;
n.s.	Not significant;
PBS	Phosphate-buffered saline;
PDAC	Pancreatic ductal adenocarcinoma;
PD-1	Programmed cell death protein 1;
PlatE	Platinum E;
PVDF	Polyvinylidene difluoride;
RBC	Red blood cell;
rEGFR	Recombinant epidermal growth factor receptor;
RT	Room temperature;
s.c.	Subcutaneous;
scFV	Single-chain variable fragment;
SDS-PAGE	Sodium dodecyl sulfate polyacrylamide gel electrophoresis;
SEM	Standard error of the mean;
siRNA	Small interfering RNA;
SPF	Specific pathogen-free;
TBS-T	Tris-buffered saline and Tween;
TCR	T cell receptor;
T_eff_	Effector T cells;
TGF- β	Transforming growth factor-β;
TIL	Tumor-infiltrating lymphocyte;
TM	Transmembrane;
TME	Tumor microenvironment;
T_naive_	Naïve T cell;
T_reg_	Regulatory T cell;
WB	Western blot;
WT	Wildtype.
