# Supplementary material for: Cblb Gene Editing in T Cells Sustains Expansion and Immunogenic CAR T Tumor Killing Under Chronic Antigenic Stimulation
Source: Cells. 2026 Aug 3;15(15):1407. doi: 10.3390/cells15151407 (PMC13464884; doi:10.3390/cells15151407)
Supplement: Supplementary file 1 [file cells-15-01407-s001.zip › cells-4439385-supplementary.pdf]

Supplementary Data

Article

Cblb gene editing in T cells sustain expansion and immunogenic CAR T tumor killing under chronic antigenic stimulation

Daniel Schreiber[1]†, Sebastian Peer[1,2]†, Christina Lutz-Nicoladoni[2,3], Jiří Koutník[1], Viktor Lang[1], Viana Wille[1], Isabel Hölzl[1], Dominik Humer[1], Nino Tokic[1], Dorothee Freimark[1], Mario Kuttke[4], Alexander Dohnal[4], Romana Gugenberger[4], Thomas Gruber[1], Nikolaus Thuille[1], Dominik Wolf[2], Victoria Klepsch[1], Kerstin Siegmund[1] and Gottfried Baier[1]*

[1] Institute for Cell Genetics, Medical University of Innsbruck, Innsbruck 6020, Tyrol, Austria
[2] Internal Medicine V, Hematology and Oncology, Comprehensive Cancer Center Innsbruck, Tyrolean Cancer
Research Institute, Medical University of Innsbruck, Innsbruck 6020, Tyrol, Austria
[3] Institute for Human Genetics, Medical University of Innsbruck, Innsbruck 6020, Tyrol, Austria
[4] invIOs GmbH, Campus-Vienna-Biocenter, Vienna 1030, Vienna, Austria
† These authors contributed equally to this work and share first authorship
* Correspondence: gottfried.baier@i-med.ac.at

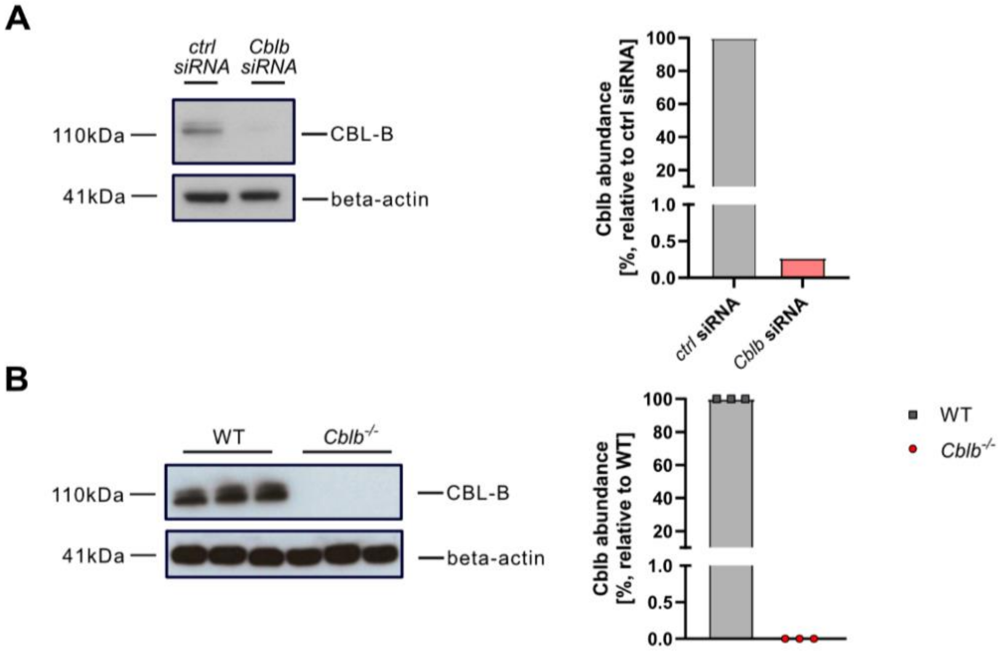

Supplementary Figure S1. Confirmation of Cblb knockdown or knockout in siRNA treated CD8+ T cells and germline CAR T cells, respectively.

(A) CBL-B protein abundance in CD8+ T cells treated with *ctrl* siRNA (left, grey) and *Cblb* siRNA (right, red.) **(B)** CBL-B protein abundance in CD8+ CAR T cells derived from WT (left, grey) and *Cblb*-deficient (right, red) mice. Exemplary immunoblots are shown. Quantification was performed using ImageJ.

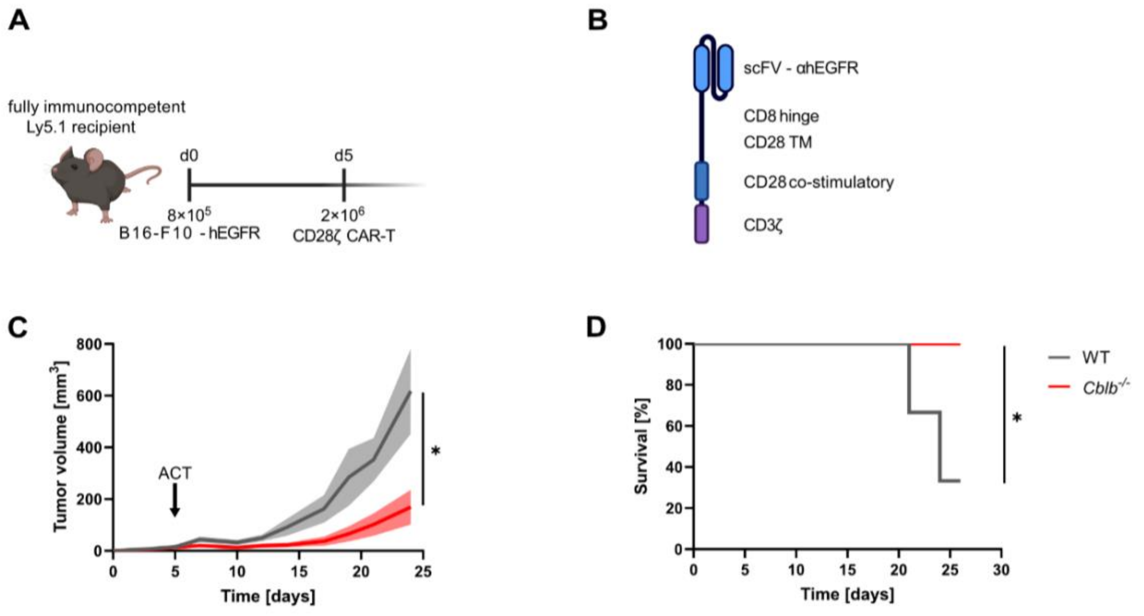

Supplementary Figure S2. Therapeutic benefit of anti-hEGFR CAR T cells in murine B16-F10 tumor model by genetic *Cblb* inhibition.

Ly5.1 mice received 8×10^5 hEGFR-expressing B16-F10 (B16-F10-hEGFR) tumor cells subcutaneously. Therapeutic ACT of 1×10^6 $CD8^+$ CAR T cells was performed on d5 after tumor injection. Tumor size and survival of mice was monitored three times a week. Length and width of each tumor was measured by using a caliper and tumor size was calculated.

(A) Schematic timeline of experiment. **(B)** Second generation CAR construct used for retroviral transduction of WT and *Cblb*$^{-/-}$ $CD8^+$ T cells. **(C)** Average tumor growth curve of one independent experiment. (n= 3-5) **(D)** Survival curve of mice, which received WT or *Cblb*$^{-/-}$ $CD8^+$ CAR-T cells. Mice harboring tumors with a size >500mm^3 were sacrificed. Statistical significance was determined using repeated-measures two-way ANOVA with Geisser-Greenhouse correction (C) and log-rank test (D) Data are presented as mean ± SEM. *$p \leq 0.05$.
